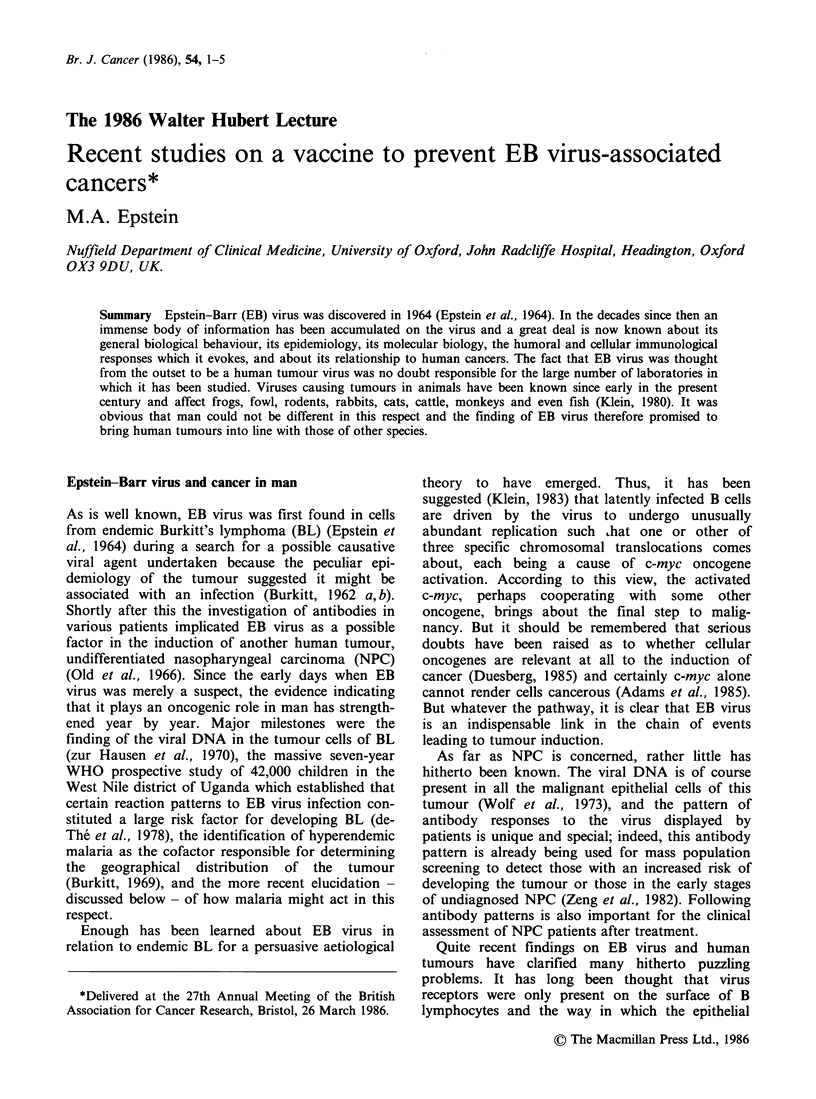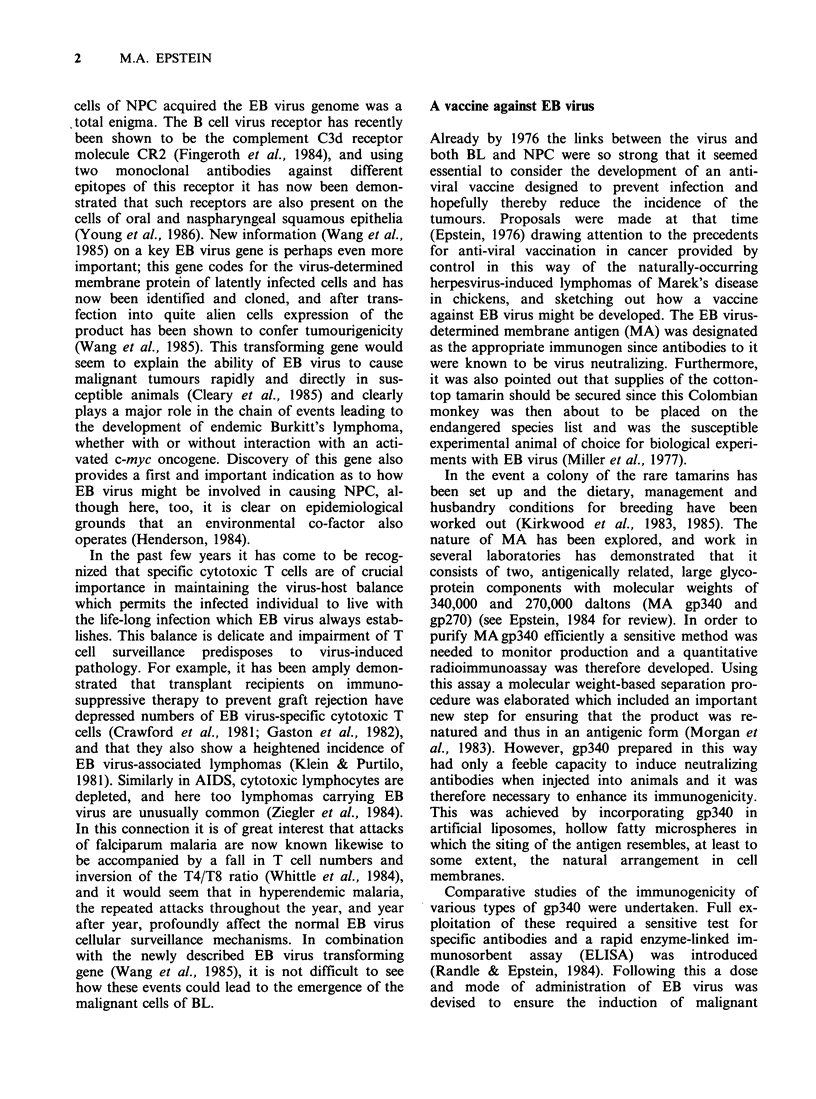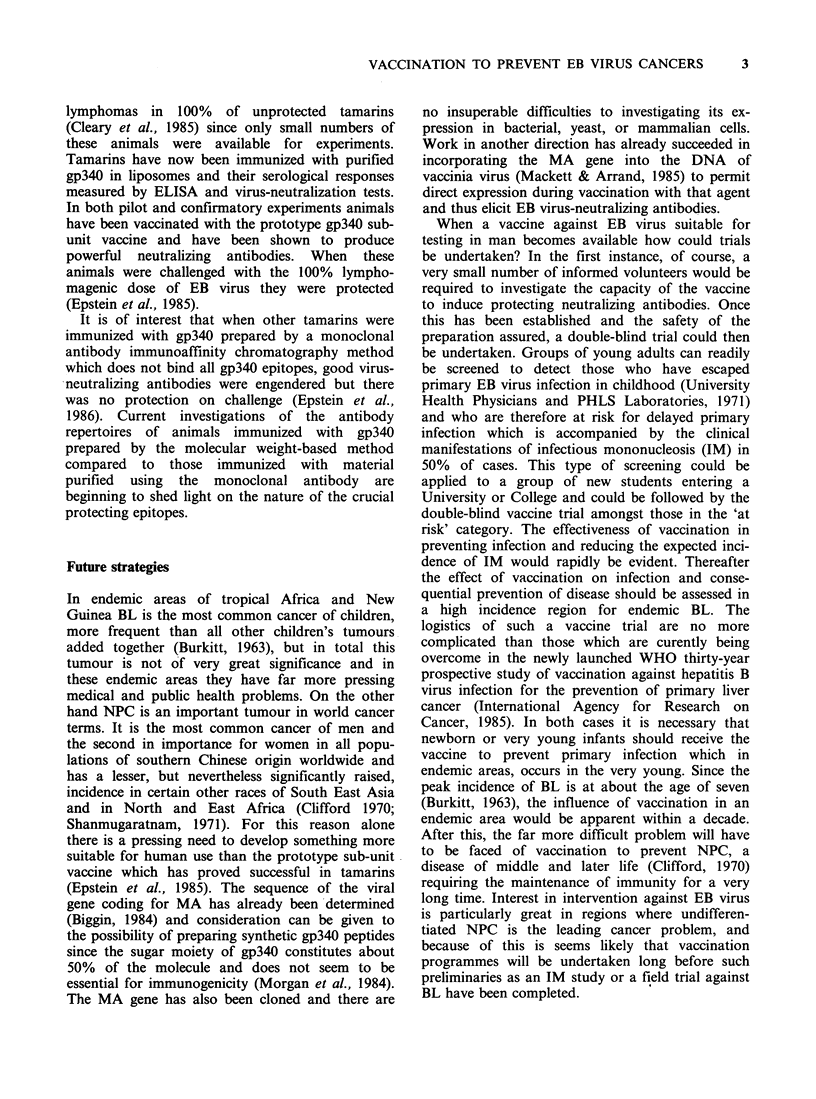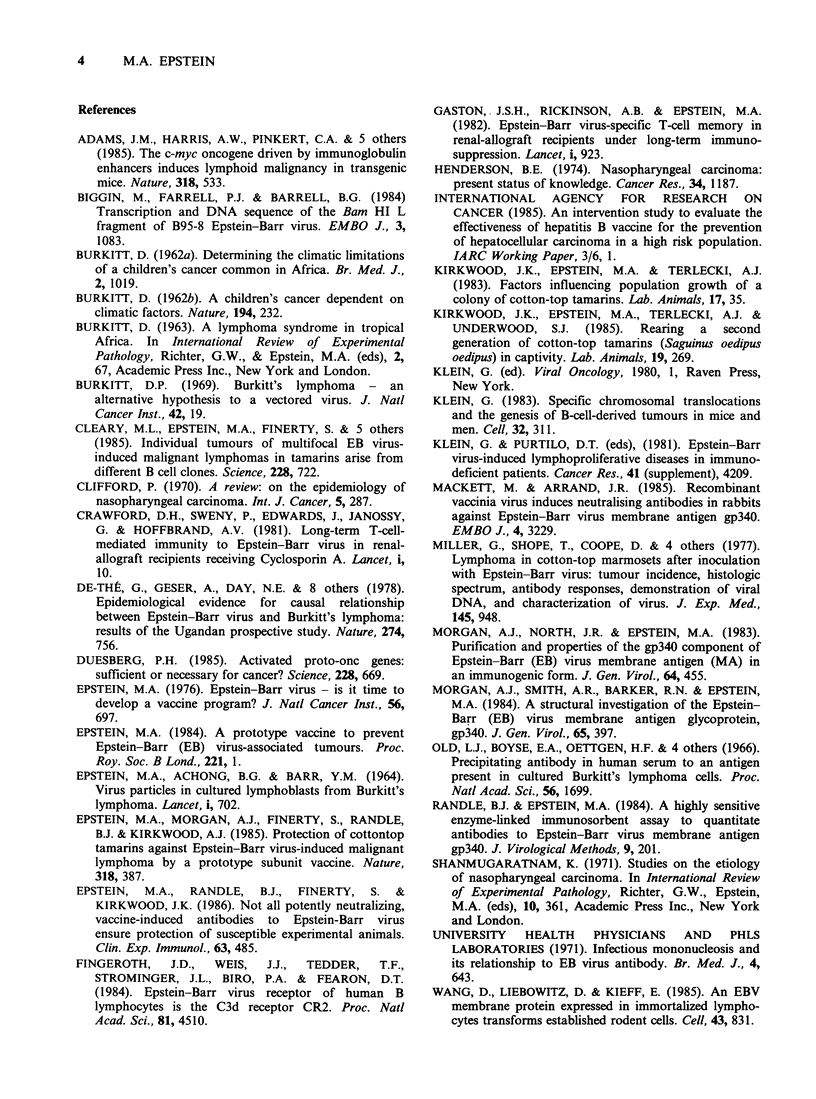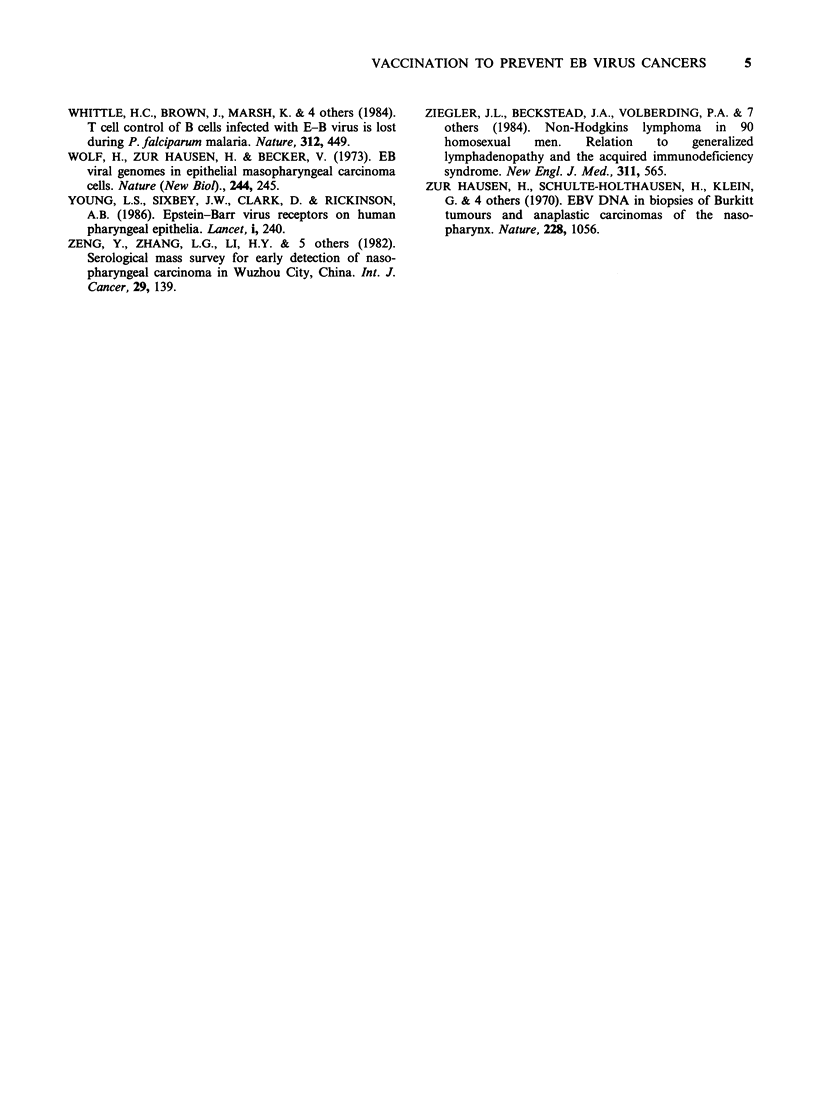# The 1986 Walter Hubert lecture. Recent studies on a vaccine to prevent EB virus-associated cancers.

**DOI:** 10.1038/bjc.1986.145

**Published:** 1986-07

**Authors:** M. A. Epstein

## Abstract

Epstein-Barr (EB) virus was discovered in 1964 (Epstein et al., 1964). In the decades since then an immense body of information has been accumulated on the virus and a great deal is now known about its general biological behaviour, its epidemiology, its molecular biology, the humoral and cellular immunological responses which it evokes, and about its relationship to human cancers. The fact that EB virus was thought from the outset to be a human tumour virus was no doubt responsible for the large number of laboratories in which it has been studied. Viruses causing tumours in animals have been known since early in the present century and affect frogs, fowl, rodents, rabbits, cats, cattle, monkeys and even fish (Klein, 1980). It was obvious that man could not be different in this respect and the finding of EB virus therefore promised to bring human tumours into line with those of other species.


Br. J. Cancer (1986), 54, 1–5

**The 1986 Walter Hubert Lecture**

### Recent studies on a vaccine to prevent EB virus-associated cancers*

M.A. Epstein

*Nuffield Department of Clinical Medicine, University of Oxford, John Radcliffe Hospital, Headington, Oxford OX3 9DU, UK.*

**Summary** Epstein–Barr (EB) virus was discovered in 1964 (Epstein *et al.*, 1964). In the decades since then an immense body of information has been accumulated on the virus and a great deal is now known about its general biological behaviour, its epidemiology, its molecular biology, the humoral and cellular immunological responses which it evokes, and about its relationship to human cancers. The fact that EB virus was thought from the outset to be a human tumour virus was no doubt responsible for the large number of laboratories in which it has been studied. Viruses causing tumours in animals have been known since early in the present century and affect frogs, fowl, rodents, rabbits, cats, cattle, monkeys and even fish (Klein, 1980). It was obvious that man could not be different in this respect and the finding of EB virus therefore promised to bring human tumours into line with those of other species.

### Epstein–Barr virus and cancer in man

As is well known, EB virus was first found in cells from endemic Burkitt's lymphoma (BL) (Epstein *et al.*, 1964) during a search for a possible causative viral agent undertaken because the peculiar epidemiology of the tumour suggested it might be associated with an infection (Burkitt, 1962 *a,b*). Shortly after this the investigation of antibodies in various patients implicated EB virus as a possible factor in the induction of another human tumour, undifferentiated nasopharyngeal carcinoma (NPC) (Old *et al.*, 1966). Since the early days when EB virus was merely a suspect, the evidence indicating that it plays an oncogenic role in man has strengthened year by year. Major milestones were the finding of the viral DNA in the tumour cells of BL (zur Hausen *et al.*, 1970), the massive seven-year WHO prospective study of 42,000 children in the West Nile district of Uganda which established that certain reaction patterns to EB virus infection constituted a large risk factor for developing BL (de-Thé *et al.*, 1978), the identification of hyperendemic malaria as the cofactor responsible for determining the geographical distribution of the tumour (Burkitt, 1969), and the more recent elucidation – discussed below – of how malaria might act in this respect.

Enough has been learned about EB virus in relation to endemic BL for a persuasive aetiological theory to have emerged. Thus, it has been suggested (Klein, 1983) that latently infected B cells are driven by the virus to undergo unusually abundant replication such that one or other of three specific chromosomal translocations comes about, each being a cause of c-*myc* oncogene activation. According to this view, the activated c-*myc*, perhaps cooperating with some other oncogene, brings about the final step to malignancy. But it should be remembered that serious doubts have been raised as to whether cellular oncogenes are relevant at all to the induction of cancer (Duesberg, 1985) and certainly c-*myc* alone cannot render cells cancerous (Adams *et al.*, 1985). But whatever the pathway, it is clear that EB virus is an indispensable link in the chain of events leading to tumour induction.

As far as NPC is concerned, rather little has hitherto been known. The viral DNA is of course present in all the malignant epithelial cells of this tumour (Wolf *et al.*, 1973), and the pattern of antibody responses to the virus displayed by patients is unique and special; indeed, this antibody pattern is already being used for mass population screening to detect those with an increased risk of developing the tumour or those in the early stages of undiagnosed NPC (Zeng *et al.*, 1982). Following antibody patterns is also important for the clinical assessment of NPC patients after treatment.

Quite recent findings on EB virus and human tumours have clarified many hitherto puzzling problems. It has long been thought that virus receptors were only present on the surface of B lymphocytes and the way in which the epithelial

*Delivered at the 27th Annual Meeting of the British Association for Cancer Research, Bristol, 26 March 1986.

© The Macmillan Press Ltd., 1986

cells of NPC acquired the EB virus genome was a total enigma. The B cell virus receptor has recently been shown to be the complement C3d receptor molecule CR2 (Fingeroth *et al.*, 1984), and using two monoclonal antibodies against different epitopes of this receptor it has now been demonstrated that such receptors are also present on the cells of oral and naspharyngeal squamous epithelia (Young *et al.*, 1986). New information (Wang *et al.*, 1985) on a key EB virus gene is perhaps even more important; this gene codes for the virus-determined membrane protein of latently infected cells and has now been identified and cloned, and after transfection into quite alien cells expression of the product has been shown to confer tumourigenicity (Wang *et al.*, 1985). This transforming gene would seem to explain the ability of EB virus to cause malignant tumours rapidly and directly in susceptible animals (Cleary *et al.*, 1985) and clearly plays a major role in the chain of events leading to the development of endemic Burkitt's lymphoma, whether with or without interaction with an activated c-*myc* oncogene. Discovery of this gene also provides a first and important indication as to how EB virus might be involved in causing NPC, although here, too, it is clear on epidemiological grounds that an environmental co-factor also operates (Henderson, 1984).

In the past few years it has come to be recognized that specific cytotoxic T cells are of crucial importance in maintaining the virus-host balance which permits the infected individual to live with the life-long infection which EB virus always establishes. This balance is delicate and impairment of T cell surveillance predisposes to virus-induced pathology. For example, it has been amply demonstrated that transplant recipients on immunosuppressive therapy to prevent graft rejection have depressed numbers of EB virus-specific cytotoxic T cells (Crawford *et al.*, 1981; Gaston *et al.*, 1982), and that they also show a heightened incidence of EB virus-associated lymphomas (Klein & Purtilo, 1981). Similarly in AIDS, cytotoxic lymphocytes are depleted, and here too lymphomas carrying EB virus are unusually common (Ziegler *et al.*, 1984). In this connection it is of great interest that attacks of falciparum malaria are now known likewise to be accompanied by a fall in T cell numbers and inversion of the T4/T8 ratio (Whittle *et al.*, 1984), and it would seem that in hyperendemic malaria, the repeated attacks throughout the year, and year after year, profoundly affect the normal EB virus cellular surveillance mechanisms. In combination with the newly described EB virus transforming gene (Wang *et al.*, 1985), it is not difficult to see how these events could lead to the emergence of the malignant cells of BL.

### A vaccine against EB virus

Already by 1976 the links between the virus and both BL and NPC were so strong that it seemed essential to consider the development of an anti-viral vaccine designed to prevent infection and hopefully thereby reduce the incidence of the tumours. Proposals were made at that time (Epstein, 1976) drawing attention to the precedents for anti-viral vaccination in cancer provided by control in this way of the naturally-occurring herpesvirus-induced lymphomas of Marek's disease in chickens, and sketching out how a vaccine against EB virus might be developed. The EB virus-determined membrane antigen (MA) was designated as the appropriate immunogen since antibodies to it were known to be virus neutralizing. Furthermore, it was also pointed out that supplies of the cotton-top tamarin should be secured since this Colombian monkey was then about to be placed on the endangered species list and was the susceptible experimental animal of choice for biological experiments with EB virus (Miller *et al.*, 1977).

In the event a colony of the rare tamarins has been set up and the dietary, management and husbandry conditions for breeding have been worked out (Kirkwood *et al.*, 1983, 1985). The nature of MA has been explored, and work in several laboratories has demonstrated that it consists of two, antigenically related, large glycoprotein components with molecular weights of 340,000 and 270,000 daltons (MA gp340 and gp270) (see Epstein, 1984 for review). In order to purify MA gp340 efficiently a sensitive method was needed to monitor production and a quantitative radioimmunoassay was therefore developed. Using this assay a molecular weight-based separation procedure was elaborated which included an important new step for ensuring that the product was renatured and thus in an antigenic form (Morgan *et al.*, 1983). However, gp340 prepared in this way had only a feeble capacity to induce neutralizing antibodies when injected into animals and it was therefore necessary to enhance its immunogenicity. This was achieved by incorporating gp340 in artificial liposomes, hollow fatty microspheres in which the siting of the antigen resembles, at least to some extent, the natural arrangement in cell membranes.

Comparative studies of the immunogenicity of various types of gp340 were undertaken. Full exploitation of these required a sensitive test for specific antibodies and a rapid enzyme-linked immunosorbent assay (ELISA) was introduced (Randle & Epstein, 1984). Following this a dose and mode of administration of EB virus was devised to ensure the induction of malignant lymphomas in 100% of unprotected tamarins (Cleary *et al.*, 1985) since only small numbers of these animals were available for experiments. Tamarins have now been immunized with purified gp340 in liposomes and their serological responses measured by ELISA and virus-neutralization tests. In both pilot and confirmatory experiments animals have been vaccinated with the prototype gp340 sub-unit vaccine and have been shown to produce powerful neutralizing antibodies. When these animals were challenged with the 100% lymphomagenic dose of EB virus they were protected (Epstein *et al.*, 1985).

It is of interest that when other tamarins were immunized with gp340 prepared by a monoclonal antibody immunoaffinity chromatography method which does not bind all gp340 epitopes, good virus-neutralizing antibodies were engendered but there was no protection on challenge (Epstein *et al.*, 1986). Current investigations of the antibody repertoires of animals immunized with gp340 prepared by the molecular weight-based method compared to those immunized with material purified using the monoclonal antibody are beginning to shed light on the nature of the crucial protecting epitopes.

### Future strategies

In endemic areas of tropical Africa and New Guinea BL is the most common cancer of children, more frequent than all other children's tumours added together (Burkitt, 1963), but in total this tumour is not of very great significance and in these endemic areas they have far more pressing medical and public health problems. On the other hand NPC is an important tumour in world cancer terms. It is the most common cancer of men and the second in importance for women in all populations of southern Chinese origin worldwide and has a lesser, but nevertheless significantly raised, incidence in certain other races of South East Asia and in North and East Africa (Clifford 1970; Shanmugaratnam, 1971). For this reason alone there is a pressing need to develop something more suitable for human use than the prototype sub-unit vaccine which has proved successful in tamarins (Epstein *et al.*, 1985). The sequence of the viral gene coding for MA has already been determined (Biggin, 1984) and consideration can be given to the possibility of preparing synthetic gp340 peptides since the sugar moiety of gp340 constitutes about 50% of the molecule and does not seem to be essential for immunogenicity (Morgan *et al.*, 1984). The MA gene has also been cloned and there are no insuperable difficulties to investigating its expression in bacterial, yeast, or mammalian cells. Work in another direction has already succeeded in incorporating the MA gene into the DNA of vaccinia virus (Mackett & Arrand, 1985) to permit direct expression during vaccination with that agent and thus elicit EB virus-neutralizing antibodies.

When a vaccine against EB virus suitable for testing in man becomes available how could trials be undertaken? In the first instance, of course, a very small number of informed volunteers would be required to investigate the capacity of the vaccine to induce protecting neutralizing antibodies. Once this has been established and the safety of the preparation assured, a double-blind trial could then be undertaken. Groups of young adults can readily be screened to detect those who have escaped primary EB virus infection in childhood (University Health Physicians and PHLS Laboratories, 1971) and who are therefore at risk for delayed primary infection which is accompanied by the clinical manifestations of infectious mononucleosis (IM) in 50% of cases. This type of screening could be applied to a group of new students entering a University or College and could be followed by the double-blind vaccine trial amongst those in the 'at risk' category. The effectiveness of vaccination in preventing infection and reducing the expected incidence of IM would rapidly be evident. Thereafter the effect of vaccination on infection and consequential prevention of disease should be assessed in a high incidence region for endemic BL. The logistics of such a vaccine trial are no more complicated than those which are curently being overcome in the newly launched WHO thirty-year prospective study of vaccination against hepatitis B virus infection for the prevention of primary liver cancer (International Agency for Research on Cancer, 1985). In both cases it is necessary that newborn or very young infants should receive the vaccine to prevent primary infection which in endemic areas, occurs in the very young. Since the peak incidence of BL is at about the age of seven (Burkitt, 1963), the influence of vaccination in an endemic area would be apparent within a decade. After this, the far more difficult problem will have to be faced of vaccination to prevent NPC, a disease of middle and later life (Clifford, 1970) requiring the maintenance of immunity for a very long time. Interest in intervention against EB virus is particularly great in regions where undifferentiated NPC is the leading cancer problem, and because of this is seems likely that vaccination programmes will be undertaken long before such preliminaries as an IM study or a field trial against BL have been completed.

**References**

ADAMS, J.M., HARRIS, A.W., PINKERT, C.A. & 5 others (1985). The c-*myc* oncogene driven by immunoglobulin enhancers induces lymphoid malignancy in transgenic mice. *Nature*, **318**, 533.

BIGGIN, M., FARRELL, P.J. & BARRELL, B.G. (1984) Transcription and DNA sequence of the *Bam* HI L fragment of B95-8 Epstein–Barr virus. *EMBO J.*, **3**, 1083.

BURKITT, D. (1962*a*). Determining the climatic limitations of a children's cancer common in Africa. *Br. Med. J.*, **2**, 1019.

BURKITT, D. (1962*b*). A children's cancer dependent on climatic factors. *Nature*, **194**, 232.

BURKITT, D. (1963). A lymphoma syndrome in tropical Africa. In *International Review of Experimental Pathology*, Richter, G.W., & Epstein, M.A. (eds), **2**, 67, Academic Press Inc., New York and London.

BURKITT, D.P. (1969). Burkitt's lymphoma – an alternative hypothesis to a vectored virus. *J. Natl Cancer Inst.*, **42**, 19.

CLEARY, M.L., EPSTEIN, M.A., FINERTY, S. & 5 others (1985). Individual tumours of multifocal EB virus-induced malignant lymphomas in tamarins arise from different B cell clones. *Science*, **228**, 722.

CLIFFORD, P. (1970). *A review*: on the epidemiology of nasopharyngeal carcinoma. *Int. J. Cancer*, **5**, 287.

CRAWFORD, D.H., SWENY, P., EDWARDS, J., JANOSSY, G. & HOFFBRAND, A.V. (1981). Long-term T-cell-mediated immunity to Epstein–Barr virus in renal-allograft recipients receiving Cyclosporin A. *Lancet*, **i**, 10.

DE-THÉ, G., GESER, A., DAY, N.E. & 8 others (1978). Epidemiological evidence for causal relationship between Epstein–Barr virus and Burkitt's lymphoma: results of the Ugandan prospective study. *Nature*, **274**, 756.

DUESBERG, P.H. (1985). Activated proto-onc genes: sufficient or necessary for cancer? *Science*, **228**, 669.

EPSTEIN, M.A. (1976). Epstein–Barr virus – is it time to develop a vaccine program? *J. Natl Cancer Inst.*, **56**, 697.

EPSTEIN, M.A. (1984). A prototype vaccine to prevent Epstein–Barr (EB) virus-associated tumours. *Proc. Roy. Soc. B Lond.*, **221**, 1.

EPSTEIN, M.A., ACHONG, B.G. & BARR, Y.M. (1964). Virus particles in cultured lymphoblasts from Burkitt's lymphoma. *Lancet*, **i**, 702.

EPSTEIN, M.A., MORGAN, A.J., FINERTY, S., RANDLE, B.J. & KIRKWOOD, A.J. (1985). Protection of cottontop tamarins against Epstein–Barr virus-induced malignant lymphoma by a prototype subunit vaccine. *Nature*, **318**, 387.

EPSTEIN, M.A., RANDLE, B.J., FINERTY, S. & KIRKWOOD, J.K. (1986). Not all potently neutralizing, vaccine-induced antibodies to Epstein-Barr virus ensure protection of susceptible experimental animals. *Clin. Exp. Immunol.*, **63**, 485.

FINGEROTH, J.D., WEIS, J.J., TEDDER, T.F., STROMINGER, J.L., BIRO, P.A. & FEARON, D.T. (1984). Epstein–Barr virus receptor of human B lymphocytes is the C3d receptor CR2. *Proc. Natl Acad. Sci.*, **81**, 4510.

GASTON, J.S.H., RICKINSON, A.B. & EPSTEIN, M.A. (1982). Epstein–Barr virus-specific T-cell memory in renal-allograft recipients under long-term immunosuppression. *Lancet*, **i**, 923.

HENDERSON, B.E. (1974). Nasopharyngeal carcinoma: present status of knowledge. *Cancer Res.*, **34**, 1187.

INTERNATIONAL AGENCY FOR RESEARCH ON CANCER (1985). An intervention study to evaluate the effectiveness of hepatitis B vaccine for the prevention of hepatocellular carcinoma in a high risk population. *IARC Working Paper*, 3/6, 1.

KIRKWOOD, J.K., EPSTEIN, M.A. & TERLECKI, A.J. (1983). Factors influencing population growth of a colony of cotton-top tamarins. *Lab. Animals*, **17**, 35.

KIRKWOOD, J.K., EPSTEIN, M.A., TERLECKI, A.J. & UNDERWOOD, S.J. (1985). Rearing a second generation of cotton-top tamarins (*Saguinus oedipus oedipus*) in captivity. *Lab. Animals*, **19**, 269.

KLEIN, G. (ed). *Viral Oncology*, 1980, 1, Raven Press, New York.

KLEIN, G. (1983). Specific chromosomal translocations and the genesis of B-cell-derived tumours in mice and men. *Cell*, **32**, 311.

KLEIN, G. & PURTILO, D.T. (eds), (1981). Epstein–Barr virus-induced lymphoproliferative diseases in immunodeficient patients. *Cancer Res.*, **41** (supplement), 4209.

MACKETT, M. & ARRAND, J.R. (1985). Recombinant vaccinia virus induces neutralising antibodies in rabbits against Epstein–Barr virus membrane antigen gp340. *EMBO J.*, **4**, 3229.

MILLER, G., SHOPE, T., COOPE, D. & 4 others (1977). Lymphoma in cotton-top marmosets after inoculation with Epstein–Barr virus: tumour incidence, histologic spectrum, antibody responses, demonstration of viral DNA, and characterization of virus. *J. Exp. Med.*, **145**, 948.

MORGAN, A.J., NORTH, J.R. & EPSTEIN, M.A. (1983). Purification and properties of the gp340 component of Epstein–Barr (EB) virus membrane antigen (MA) in an immunogenic form. *J. Gen. Virol.*, **64**, 455.

MORGAN, A.J., SMITH, A.R., BARKER, R.N. & EPSTEIN, M.A. (1984). A structural investigation of the Epstein–Barr (EB) virus membrane antigen glycoprotein, gp340. *J. Gen. Virol.*, **65**, 397.

OLD, L.J., BOYSE, E.A., OETTGEN, H.F. & 4 others (1966). Precipitating antibody in human serum to an antigen present in cultured Burkitt's lymphoma cells. *Proc. Natl Acad. Sci.*, **56**, 1699.

RANDLE, B.J. & EPSTEIN, M.A. (1984). A highly sensitive enzyme-linked immunosorbent assay to quantitate antibodies to Epstein–Barr virus membrane antigen gp340. *J. Virological Methods*, **9**, 201.

SHANMUGARATNAM, K. (1971). Studies on the etiology of nasopharyngeal carcinoma. In *International Review of Experimental Pathology*, Richter, G.W., Epstein, M.A. (eds), **10**, 361, Academic Press Inc., New York and London.

UNIVERSITY HEALTH PHYSICIANS AND PHLS LABORATORIES (1971). Infectious mononucleosis and its relationship to EB virus antibody. *Br. Med. J.*, **4**, 643.

WANG, D., LIEBOWITZ, D. & KIEFF, E. (1985). An EBV membrane protein expressed in immortalized lymphocytes transforms established rodent cells. *Cell*, **43**, 831.

WHITTLE, H.C., BROWN, J., MARSH, K. & 4 others (1984). T cell control of B cells infected with E–B virus is lost during *P. falciparum* malaria. *Nature,* **312,** 449.

WOLF, H., ZUR HAUSEN, H. & BECKER, V. (1973). EB viral genomes in epithelial masopharyngeal carcinoma cells. *Nature* (*New Biol*)., **244,** 245.

YOUNG, L.S., SIXBEY, J.W., CLARK, D. & RICKINSON, A.B. (1986). Epstein–Barr virus receptors on human pharyngeal epithelia. *Lancet,* **i,** 240.

ZENG, Y., ZHANG, L.G., LI, H.Y. & 5 others (1982). Serological mass survey for early detection of nasopharyngeal carcinoma in Wuzhou City, China. *Int. J. Cancer,* **29,** 139.

ZIEGLER, J.L., BECKSTEAD, J.A., VOLBERDING, P.A. & 7 others (1984). Non-Hodgkins lymphoma in 90 homosexual men. Relation to generalized lymphadenopathy and the acquired immunodeficiency syndrome. *New Engl. J. Med.,* **311,** 565.

ZUR HAUSEN, H., SCHULTE-HOLTHAUSEN, H., KLEIN, G. & 4 others (1970). EBV DNA in biopsies of Burkitt tumours and anaplastic carcinomas of the nasopharynx. *Nature,* **228,** 1056.